# Transactions of the Illinois State Medical Society

**Published:** 1853-11

**Authors:** 


*Transactions of the Illinois State Medical Society.* June, 1853.

THE volume of transactions for the present year contains, besides the minutes of the annual meeting, the annual address before the Society by Dr. Davis, an interesting and valuable report on Drugs and Medicines by Dr. Blaney, together with papers contributed by Drs. Thompson, Hall and Andrews.

The address by Prof. Davis will be read with pleasure. It demonstrates the intimate relation of medicine to the whole range of natural sciences, while in its practice it is shown to be closely interwoven with all social interests of society. Dr. Davis has labored earnestly and with a will, for the advancement of true medical science, for the elevation of the standard of professional education, and in his address before the State Society, he urges as a means for the accomplishment of this end, the recognition of individual responsibility. It is shown that the error lies at the commencement of the course of study. Physicians receive into their offices young men whose preliminary education is poor; after a short curriculum of study they commence practice, some without even having attended a medical college, others after having listened to a single course of lectures, while comparatively a few appreciating the value of a thorough professional education, are content to toil that they may finally reap the rewards of toil, to carefully study nature, that they may be prepared as her priests to interpret her mysteries.

The report of the committee on Drugs and Medicines briefly exhibits the condition of Pharmacy in the United States at the present time.

A note of inquiry was addressed to the different druggists of this city. In reply to the several questions propounded the following answers were obtained:

"1st. The law of 1848 for the inspection of drugs, &c., has operated decidedly upon the drug market, and to the great improvement of the quality of many articles, particularly roots, such as Rhubarb, Sarsaparilla, and Jalap; barks, particularly Cinchona, and Gum Opium.

The law has not fully accomplished the object for which it was designed, but has answered all reasonable expectations. Its good results have been slow; for, the drug condemned in one port, has been repeatedly re-shipped to hail from some unexpected point, and perhaps find entry at some small port unwatched by the inspectors; but continued vigilance has increased the risk and cost of such shipments, until the *worthless* articles are as scarce as the *really good* four years since; indeed, they are not in first hands at all, although considerable yet remains in the hands of retailers.

2d We have noticed a decided improvement in the quality of drugs and medicines of home production since the operation of the Drug Law.

3d. We have abundant reason to suppose home adulteration is practised to a great extent, and Vol. Oils, Powdered Roots, Barks, and Gums, are the prominent subjects of it. Cream of Tartar is very extensively adulterated, and careful large dealers purchase crystals of this and most other articles of this character, and have them ground as the only way of securing them positively pure.

There are extensive operators who make the adulteration of oils a regular trade, and all false barks and roots that most resemble genuine, are in active demand for adulterations, and bring a price proportionate to their resemblance to officinal kinds.

4th. Medicinal preparations and substances of home manufacture and productions are generally of uniform and good quality, but we think American manufacturers fail in the quality of Iodine, Iodide of Potash, Precip. Carb. Iron, and some other preparations of Iron, Nit. Silver, Spts. Nit. Dulc., Aq. Ammon., Ethers, and Chloroform, all of which are variable in quality, and some of them never good.

The coarser substances used largely in the arts, and chiefly made for that end, such as Nit. Potash, Sulph. Iron, Pruss. Blue, &c., are often used medicinally, but are so entirely unfitted by their impurity, that the chemically pure ought to be carefully selected for prescription use.

5th. American Quinine has been of unexceptionable quality for four years past, and honest in weight and quality.

Sulph. Morphine has generally been good, but of more variable quality than Quinine. A large share of the Blue-mass, made here, is very perfectly manufactured and of reliable strength, and this is true of all the preparations of mercury.

Rhubarb root, Ipecac in root and powder, are generally good in first hands. There are large quantities of miserable extracts in market, but extracts of most excellent quality are made by two American Houses, (we refer to Messrs. Schieffelen, Haines & Co., and Tilden & Co.,) and are much more reliable than most of the imported.

6th. *It is* "usual with dealers, in this city, to keep on sale articles of different quality and strength," such as all powders of Gums, Roots, Barks, Ether, Aq. Ammon, Spts. Nit. Dulc., Nit. Silver and Extracts.

We are obliged to say Physicians *do not* "generally buy the 'purest and best' qualities," although the proportion that do increases encouragingly.

Physicians and country dealers generally, buy the inferior or medium qualities, to use and to sell, because the price is lower.—Families in the city generally purchase the *best* without particular reference to price. This is particularly the case with Americans and English of the best class.

7th. Officinal preparations are *not* always made "according to the U. S. P. or of the purest and best materials." There is, however, more lack of the proper attention to *quality* than *quantity*, and therefore cannot be of uniform strength. This is a branch of this subject, of very great importance, and calls for as much action as any part of it. A large share of the compounds and drugs dispensed in the retail shops are outrageous in their quality, and the compounds not only made from worthless material, but put together by boys or incompetent hands.

The purchasers are the country practitioner and the unfortunate community at large. These remarks apply more particularly to country shops, although those of the city are, unfortunately, more or less owned or conducted by uneducated apothecaries.

8th. We have frequently seen samples of Laudanum and Paregoric, made and sold in this city, so abominably compounded as to scarcely resemble a good preparation, and almost entirely inert.

They are sometimes dispensed to families, and sometimes bottled for the jobbing trade.

9th. Foreign Quinine has not been sold in this market to any considerable extent for several years."

From these replies it will be seen that physicians are to a certain extent, guilty of encouraging the adulteration of drugs by purchasing those of inferior quality and of lower price. This is a humiliating confession, wrung from us by the facts brought forward by the chairman of the committee. The report is a valuable addition to the literature of our State, and reflects honor alike on its author and the Society.

The contributions by Drs. Hall and Andrews have already been published in this Journal. The article of Dr. Thompson we shall refer to again. J.

